# Short-Term Time Trends in Prescribing Therapy for Hypothyroidism: Results of a Survey of American Thyroid Association Members

**DOI:** 10.3389/fendo.2019.00031

**Published:** 2019-01-30

**Authors:** Jacqueline Jonklaas, Eshetu Tefera, Nawar Shara

**Affiliations:** ^1^Division of Endocrinology, Georgetown University, Washington, DC, United States; ^2^Department of Biostatistics and Biomedical Informatics, Medstar Health Research Institute, Hyattsville, MD, United States

**Keywords:** hypothyroidism, combination therapy, liothyronine, thyroid extract, trends over time

## Abstract

**Objective:** Hypothyroid patients frequently request specific therapies from their physicians. Combination therapy is vigorously discussed at professional meetings. We wished to determine if physician prescribing patterns for hypothyroidism changed during 2017 after specific educational events.

**Methods:** A survey addressing treatment of hypothyroidism was emailed to American Thyroid Association (ATA) members on three occasions in 2017. The Spring emails were sent prior to a satellite symposium addressing hypothyroidism, and prior to the annual Endocrine Society and ATA meetings; the December emails were sent after these events. Physicians were presented with thirteen theoretical patients and chose from 6 therapeutic options, including levothyroxine, synthetic combination therapy, thyroid extract, and liothyronine monotherapy. The patient scenarios successively incorporated factors potentially providing reasons for considering combination therapy. Multivariate repeated measures logistic regression analyses first examined effects of physician characteristics on prescribing the various therapies. Then, analyses also incorporated timing, by comparing prescribing patterns in February, March, and December.

**Results:** In analyses of prescribing levothyroxine monotherapy vs. any T3 therapy, there was a trend of borderline significance (*p* = 0.053) for T3 therapy to be prescribed more in December compared with February-March combined. When multivariate analyses were performed controlling for time and physician characteristics, choice of therapy was only significantly affected by country of practice (OR 1.7, CI 1.3–2.2). Physician choice of therapies was also examined for the options of continuing (1) levothyroxine, vs. (2) increasing levothyroxine, (3) adding liothyronine either with or without levothyroxine reduction, or (4) replacing levothyroxine with desiccated thyroid extract or liothyronine. When multivariate analyses incorporating time and physician characteristics were performed, respondents in December (OR 1.5, CI 1.0–2.3) and those practicing in North America (OR 1.8, CI 1.2–2.6) were more likely to prescribe liothyronine.

**Conclusions:** This survey shows that although current North American guidelines do not recommend combination therapy, such therapy is being prescribed more over time and is also more commonly prescribed in North America. It is possible our guidelines are failing to incorporate evidence that physicians are considering when prescribing combination therapy. Such evidence could include data about patient preferences, and this needs to be a focus of future studies.

## Introduction

Society guidelines concerning the treatment of hypothyroidism originate from both North America and Europe, and have been published during a time period spanning 2012–2016. In 2012 one guideline from Europe (1), one from North America (2) and one narrative review authored by American and European experts (3) were published. The European guidelines (1) and the narrative review (3) both suggested that therapy combining levothyroxine (LT4) and liothyronine (LT3) could be considered under certain specific circumstances, whereas the co-authored 2012 American Association of Clinical Endocrinologists (AACE)/ American Thyroid Association (ATA) guidelines (2) did not recommend combination therapy. Updated ATA Guidelines for the Treatment of Hypothyroidism were published in 2014 (4). These guidelines concluded that there was insufficient evidence to recommend combination therapy (4).

The majority of the clinical studies of synthetic combination therapy upon which the various guidelines have based their recommendations were published between 1999 and 2009 (5–17). A single trial of therapy with desiccated thyroid extract was published in 2013 (18). Since the publication of the 2014 guidelines, one additional original research study, which did not identify an advantage of combination therapy has been published (19). In addition, subsequently published British Thyroid Association guidelines (20) have suggested that combination therapy could be prescribed and carefully monitored under certain circumstances. Most recently, the Italian Endocrine Society and the Italian Association of Clinical Endocrinologists have also suggested that combination therapy could be considered (21, 22). None of these guidelines have supported the use of desiccated thyroid extract (DTE).

From a consideration of the various guidelines, it appears that rather than seeing a trend over time toward encouraging or discouraging combination therapy, there has simply been a fluctuation in time. However, it does appear that European guidelines, generally authored by physicians practicing in Europe, have favored consideration of combination therapy. At the present, other than the one additional clinical study already mentioned (19), there have not been additional randomized clinical trials of combination therapy that might potentially alter physician prescribing. However, some studies about patient preference have been added to the literature (23–25). Previous studies from our group have shown that patient and physician characteristics affect the tendency to prescribe combination therapy (26, 27). The goal of this particular analysis was to assess the effect of short term time trends in prescribing patterns. This report compares the prescribing pattern of physicians surveyed in early 2017 and then again in late 2017. Intervening in between these two deployments of the survey was an ATA satellite symposium dedicated to the treatment of hypothyroidism that was offered prior to the 2017 annual Endocrine Society meeting, the Endocrine Society meeting itself, and the 2017 annual meeting of the ATA.

## Methods

### Survey Content and Distribution

This survey of ATA members was designed to determine their choice of therapy for hypothyroidism when presented with several different theoretical patients. The study was approved by the Georgetown University Institutional Review Board and the survey questions are included as Supplemental Material. A link for the survey was distributed to all ATA members via email on several occasions in 2017. The survey link was distributed in February 2017, March 2017, and December 2017. The introduction to the first survey deployment outlined the goals of the survey and explained that the survey would be distributed again after key professional meetings had occurred. The introduction to the March survey had the same explanation, but stressed that those who had responded in February need not take the survey again. The two December deployments of the survey reiterated its goals and specifically invited those who had responded previously to re-take the survey.

The index patient was a 29-year old female with Hashimoto's hypothyroidism who had no specific complaints while taking LT4 replacement therapy. Her vital signs were normal and her body mass index was 25 kg/m2. She was described as having overt hypothyroidism of at least 5 years duration, being compliant with therapy, and not considering pregnancy. Her biochemical assessment showed a thyroid stimulating hormone (TSH) value of 2.2 mIU/L (normal range 0.4–4.0 mIU/L), a free thyroxine (FT4) value of 1.3 ng/dL (normal range 0.8–1.8 ng/dL), and a triiodothyronine (T3) value of 120 ng/dL (normal range 80–180 ng/dL). Twelve additional patient scenarios then introduced factors that have been suggested in the literature to potentially provide reasons for considering combination therapy. Examples of these factors included presence of symptoms, low serum T3 concentration, a patient request for T3, documentation of deiodinase polymorphism status (28–30) etc (see Table 1), table also included in prior reports (26, 27). Survey respondents were asked to select from the following treatment options for each of the 13 patient scenarios presented: (a) Continue current levothyroxine, (b) Increase levothyroxine dose, (c) Add 2.5 mcg liothyronine (Cytomel) twice daily and reduce levothyroxine, (d) Add 2.5 mcg liothyronine (Cytomel) twice daily to current levothyroxine, (e) Replace levothyroxine with thyroid extract (e.g., armor thyroid), (f) Replace levothyroxine with liothyronine (Cytomel) as single therapy (see left hand columns of Tables 2A,B).

**Table 1:** Patient characteristics in questions 5–17^*^.

**(A) Patient characteristics**	**Characteristics present in question stem according to question number**												
	**Q5**	**Q6**	**Q7**	**Q8**	**Q9**	**Q10**	**Q11**	**Q12**	**Q13**	**Q14**	**Q15**	**Q16**	**Q17**
Symptoms	No	Yes	Yes	Yes	Yes	Yes	Yes	Yes	Yes	Yes	Yes	Yes	Yes
Serum TSH (mIU/L)	2.2	2.2	3.9	2.2	3.9	2.2	2.2	2.2	2.2	2.2	2.2	2.2	2.2
Serum T3 (ng/dL)	120	120	120	75	75	75	75	75	75	75	75	75	75
Requests LT3	No	No	No	No	No	Yes	Yes	Yes	Yes	Yes	Yes	Yes	Yes
Athyreotic	No	No	No	No	No	No	Yes	No	No	No	No	No	No
LT3 preference	No	No	No	No	No	No	No	Yes	No	No	No	No	No
Male	No	No	No	No	No	No	No	No	Yes	No	No	No	No
Polymorphism	No	No	No	No	No	No	No	No	No	Yes	No	No	No
Age	29	29	29	29	29	29	29	29	29	29	59	29	59
BMI	25	25	25	25	25	25	25	25	25	25	25	32	25
Comorbidity	No	No	No	No	No	No	No	No	No	No	No	No	Yes

* *Question numbers refer to those used in the survey provided in the Supplementary Material. Each question incorporates the patient characteristics in the left-hand column, as indicated by each column in the body of the table*.

**Table 2A:** Response to questions regarding therapy (Q5–Q10).

**Percentage of respondents choosing each treatment option**						
Question #	Q5	Q6	Q7	Q8	Q9	Q10
Patient characteristics	Feels well, TSH 2.2, T3 120	Sxs, TSH 2.2, T3 120	Sxs, TSH 3.9, T3 120	Sxs, TSH 2.2, T3 75	Sxs, TSH 3.9, T3 75	Sxs, request, TSH 2.2, T3 75
**Treatment Options**	**Feb**					
Continue LT4	97.56	61.85	23.25	45.20	14.23	32.40
Increase LT4	1.22	18.88	69.52	17.60	64.08	10.80
Add LT3, ↓LT4	0.41	11.24	0.40	17.60	2.81	32.00
Add LT3 to LT4	0.41	6.02	5.62	16.00	17.27	17.60
Switch to DTE	0.41	1.61	1.20	3.60	1.20	7.20
LT3 only	0.00	0.40	0.00	0.00	0.40	0.00
**Treatment Options**	**Mar**					
Continue LT4	98.25	61.06	21.18	42.60	15.79	30.97
Increase LT4	1.75	18.58	69.79	21.16	62.88	7.96
Add LT3, ↓LT4	0.00	12.39	2.02	20.46	3.26	38.05
Add LT3 to LT4	0.00	7.08	7.02	13.16	17.19	18.58
Switch to DTE	0.00	0.88	0.00	2.63	0.88	4.42
LT3 only	0.00	0.00	0.00	0.00	0.00	0.00
**Treatment Options**	**Dec**					
Continue LT4	97.66	50.39	11.72	34.13	10.16	24.60
Increase LT4	2.34	25.20	72.91	23.02	59.28	11.11
Add LT3, ↓LT4	0.00	16.54	2.91	20.63	7.91	36.51
Add LT3 to LT4	0.00	6.30	9.47	19.84	21.88	24.60
Switch to DTE	0.00	1.57	0.00	1.59	0.78	3.17
LT3 only	0.00	0.00	0.00	0.79	0.00	0.00

**Table 2B:** Response to questions regarding therapy (Q11–Q17).

	**Percentage of Respondents Choosing Each Treatment Option**						
Question #	Q11	Q12	Q13	Q14	Q15	Q16	Q17
Patient characteristics	Sxs, request, thyX, TSH 2.2, T3 75	Sxs, request, prior LT3, TSH 2.2, T3 75	Sxs, request, male, TSH 2.2, T3 75	Sxs, request, polym, TSH 2.2, T3 75	Sxs, request, 59 yo, TSH 2.2, T3 75	Sxs, request, BMI 32, TSH 2.2, T3 75	Sxs, request, co-morb, TSH 2.2, T3 75
**Treatment Options**	**Feb**					
Continue LT4	27.71	25.40	31.73	15.60	39.36	28.80	47.20
Increase LT4	14.06	4.60	13.25	8.00	10.44	12.80	10.00
Add LT3, ↓LT4	31.33	42.80	30.92	40.80	34.14	30.40	30.00
Add LT3 to LT4	21.29	22.00	18.88	27.20	10.84	22.00	8.00
Switch to DTE	5.62	5.20	5.22	4.00	5.22	5.60	4.40
LT3 only	0.00	0.00	0.00	4.40	0.00	0.40	0.40
**Treatment Options**	**Mar**						
Continue LT4	28.95	27.43	30.70	18.58	38.94	31.58	46.49
Increase LT4	10.53	6.19	9.65	4.42	12.39	10.53	10.53
Add LT3, ↓LT4	33.33	38.05	38.60	43.36	31.86	34.21	28.07
Add LT3 to LT4	21.93	25.66	17.54	31.86	12.39	19.30	10.53
Switch to DTE	5.26	2.65	3.51	1.77	4.42	4.39	4.39
LT3 only	0.00	0.00	0.00	0.00	0.00	0.00	0.00
**Treatment Options**	**Dec**						
Continue LT4	18.75	19.53	21.88	14.17	28.35	21.88	41.41
Increase LT4	17.19	6.25	13.28	2.36	10.24	15.63	7.81
Add LT3, ↓LT4	38.28	46.88	38.28	43.31	45.67	34.38	35.16
Add LT3 to LT4	24.22	27.34	25.00	37.80	14.17	26.56	13.28
Switch to DTE	1.56	0.00	1.56	1.57	1.57	1.56	2.34
LT3 only	0.00	0.00	0.00	0.79	0.00	0.00	0.00

### Statistical Analysis

The goals of the survey were to determine whether (i) patient characteristics and (ii) physician characteristics affected choice of therapy for patients (26, 27), and (iii) whether these choices changed over time. The goal of this particular analysis was to determine whether these choices changed over time. The results of the survey are initially presented as the percentage of survey respondents selecting each therapeutic option for the 13 different patient scenarios. Two different treatments of the data were then applied. The first was a binary analysis examining whether a respondent would prescribe LT4 vs. any therapy other than LT4. The second examined the prescribing choice with the therapies categorized into four groups (1–4).

For the binary analysis, repeated measures logistic regression analysis was used to examine the relationship between the treatment chosen and patient and physician characteristics, and between physician characteristics and time of the survey (February compared with March, February compared with December, and February-March compared with December). The February vs. March comparison was performed as an internal control, as no change would be expected in this time period. Choice of either continuing or increasing LT4 (options a or b) was used as the reference and compared with choice of anything other than LT4 (choices c, d, e and f from the prescription options). The method of generalized estimating equations (GEE) was used to account for correlations among the 13 responses from the same physician. Multivariate repeated measures logistic regression analysis was also conducted controlling for patient and physician characteristics, and physician characteristics and time of the survey.

For the second analysis the response options were grouped into 4 groups as follows: group 1: continue LT4 (option a), group 2: increase LT4 (option b), group 3: add 2.5 mcg liothyronine both with or without LT4 reduction (options c and d), and group 4: replace LT4 with DTE or LT3 (options e and f). The grouping of the response options was utilized due to the small numbers of these options chosen for some patient scenarios. The choice to continue current LT4 was used as the reference. Repeated measures multinomial logistic regression analysis was used to adjust for correlations among responses from the same physician, while examining the relationship between the therapy chosen and patient and physician characteristics, and between physician characteristics and time of survey [February (reference) vs. March, February vs. December, and February-March vs. December]. The February vs. March comparison was again performed as an internal control, as no change would be expected in this time period. Multivariate repeated measures multinomial logistic regression analysis was also conducted controlling for patient and physician characteristics, and between physician characteristics and time of survey.

For both analyses, odds ratios with corresponding 95% confidence intervals and *p*-values were calculated. Statistical significance was defined as *P* < 0.05. *P*-values of between 0.05 and 1.0 were considered as trendwise or of borderline significance. An in-depth analysis of the effect of patient and physician characteristics has been reported (26, 27).

## Results

### Physician Respondents

There were 249, 114, and 128 eligible responses to the survey from physicians who routinely prescribed therapy for hypothyroidism in February, March, and December of 2017, respectively. IP addresses were used to ensure that there were no duplicate responses in February and March, and that all December respondents had previously answered the survey in the Spring.

The responses rates from the 1,798 members of the ATA in 2017 are 14, 6.3, and 7.1%, respectively. Thus, there were 363 responses to the survey when it was initially deployed in February-March and 128 responses to the December deployment. The responding physicians were 83–88% endocrinologists, 58–75% were from North America and 12–20% were from Europe. Seventeen-twenty five percent had been in practice for 11–20 years, and 49–62% had been in practice for more than 20 years (see Table 3).

**Table 3:** Characteristics of physicians responding to survey.

**Question regarding physician characteristic**	**Response options**	**% at Survey time point 1**	**% at Survey time point 2**	**% at Survey time point 3**	**% ATA composition in 2017**
Do you prescribe and adjust LT4 therapy for patients with hypothyroidism?	Yes	100^*^	100^*^	100^*^	-
	No	0	0	0	-
How many years have you been in practice?	In training	2.8	2.6	1.6	-
	<5 years	9.2	7.9	7.8	-
	5–10 years	14.0	9.7	11.7	-
	11–20 years	24.8	17.5	21.1	-
	>20 years	49.20	62.3	57.8	-
Where do you practice?	North America	58.4	74.6	73.4	74
	South America	7.6	4.4	1.6	3
	Europe	20	12.3	15.6	9
	Asia	8.4	7.0	7.0	12
	Other	5.6	1.8	2.3	1
Which best describes your specialty?	Endocrinologist	87.6	82.5	83.6	63
	Surgeon	4.8	4.4	8.6	18
	Nuclear Medicine Physician	3.2	6.1	3.9	3
	Internist or Primary Care Physician	1.2	2.6	0.8	17
	Other	3.2	4.4	3.1	

* *results only reported for those who answered yes*.

### Descriptive Findings for the Patient Scenarios

**Figure 1:**
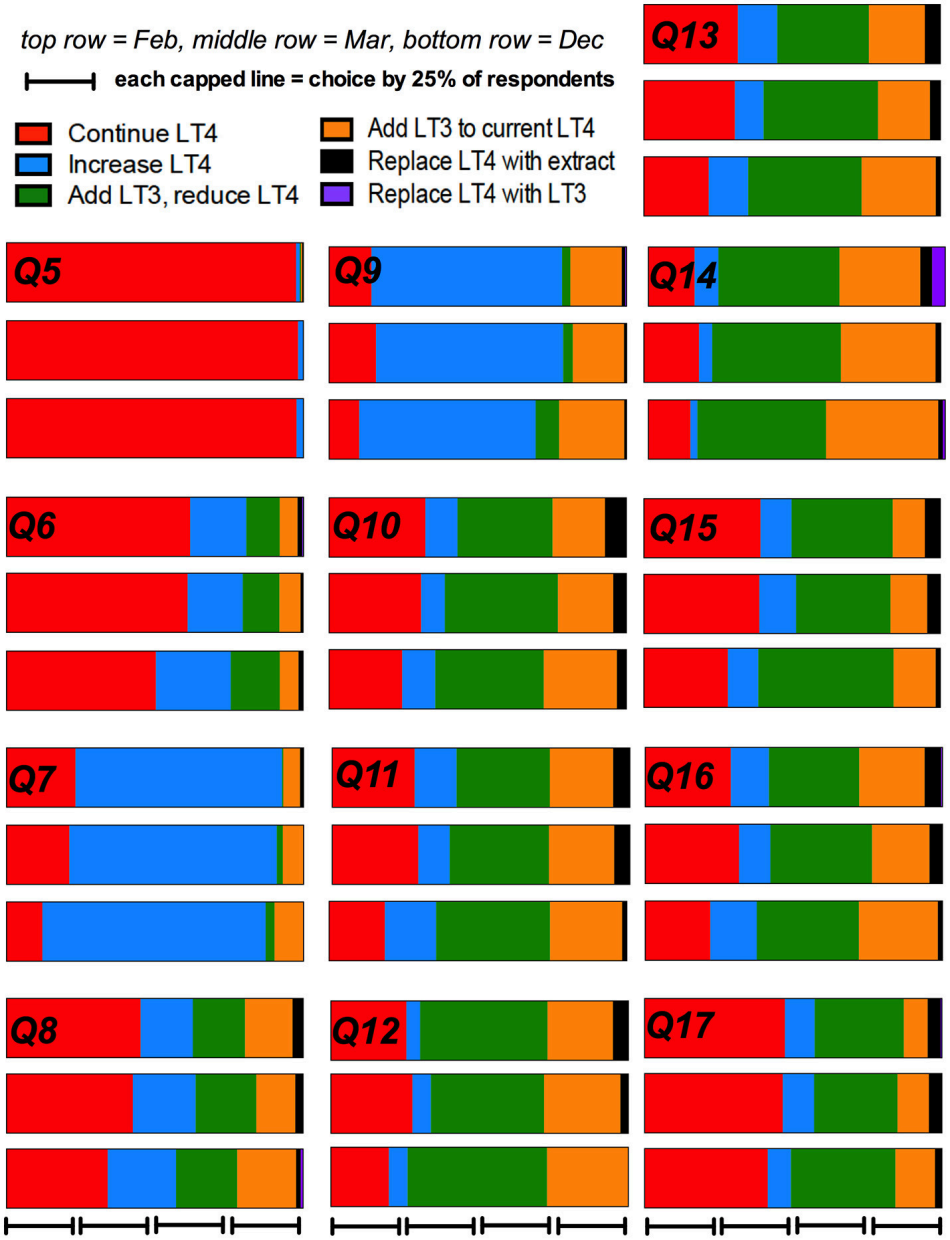
Response to patient scenarios over time.

The percentage of physician respondents choosing each individual treatment option at each of the three time points broken down by the 13 different patient scenarios is shown in Tables 2A,B. These data are also displayed graphically in Figure 1.

### Patient and Physician Characteristics

#### Analysis With Binary Therapeutic Options for the 363 First Time Respondents

Multivariate repeated measures logistic regression analysis was conducted to control for all patient and physician characteristics. Patient symptoms, T3 levels, TSH levels, presence of a polymorphism, request for T3 therapy, and a stated preference for T3 therapy made it more likely that a physician would prescribe a therapy other than LT4 monotherapy (i.e., T3-containing therapy), with a *p* < 0.0001 in each case. Older age and presence of a comorbidity made it significantly more likely the physician would prescribe LT4 (*p* < 0.0001 and 0.0002, respectively). With respect to physician characteristics only country of practice affected prescribing pattern. Physicians practicing in North America were more likely to prescribe therapy other than LT4, compared with physicians from other regions (*p* < 0.0001). (see Tables 4, 3b respectively) in previously published reports (26, 27).

#### Analysis With Multiple Therapeutic Options for the 363 First Time Respondents

When multivariate logistic regression analyses were performed to determine whether patient characteristics affected whether physicians would prescribe continued LT4 (group 1 option) vs. increasing LT4 (group 2 option) vs. adding LT3 to the same or reduced LT4 (group 3 options) vs. replacing LT4 with T3-containing therapy comprised of either DTE or LT3 (group 4 options) most patient characteristics (patient symptoms, T3 levels, TSH levels, presence of a polymorphism, request for T3 therapy, and a stated preference for T3 therapy) appeared to be significant in the model (*p* < 0.0001). Older age and presence of a comorbidity made it significantly more likely the physician would continue LT4 (*p* < 0.0002 and 0.04, respectively). When multivariate analyses of physician characteristics were performed only country of practice was significant, with physicians practicing in North America being more likely to add LT3 to LT4 (OR 1.9, CI 1.2–2.9) and more likely to prescribe DTE or LT3 monotherapy (OR 1.7, CI 1.0–2.9). (see Tables 6, 4b, respectively) as previously published reports (26, 27).

### Trends Over Time

#### Analysis With Binary Therapeutic Options

The prescribing trends over time are shown graphically in Figure 1. In univariate analysis of the binary option of prescribing LT4 vs. other therapies, a comparison of the results from respondents who completed the survey in Feb and March, compared with those who completed the survey in December showed there was a non-significant trend for physicians to prescribe therapy other than LT4 (OR 1.28, 95% CI 0.99–1.65, *p* = 0.053) (see Table 4). Comparison of February with March and then February with December showed OR 1.01, 95% CI 0.76–1.34, *p* = 0.95, and OR 1.29, 95% CI 0.99–1.69, *p* = 0.062, respectively. Additionally, the physician country of practice appeared to have a significant effect on choice of therapy (OR 1.7, CI 1.4–2.2, *p* < 0.0001). However, when multivariate analysis was performed controlling for time (Dec vs. Feb-Mar), and physician characteristics, choice of therapy was only significantly affected by country of practice (OR 1.7, CI 1.3–2.2, *p* < 0.0001), and time no longer showed a trend.

**Table 4:** Univariate analyses of the effect of timing on physician prescribing of LT4 vs. any T3-containing therapy.

**Time**	**Odds ratio**	**95% Confidence interval**		***P*-value**
March (Feb = ref)	1.01	0.76	1.34	0.9531
Dec (Feb = ref)	1.29	0.99	1.69	0.0616
December (Feb-Mar = ref)	1.28	0.99	1.65	0.053

#### Analysis With Multiple Therapeutic Options

Physician choice of therapies over time was examined for the grouped options of (1) continuing LT4 (option a), vs. either (2) increasing LT4 (option b), (3) adding 2.5 mcg liothyronine either with or without LT4 reduction (options c and d), and (4) replacing LT4 with DTE or LT3 (options e and f). In univariate analyses in which the February survey results were used as the reference and compared with the responses in March and December, physicians were more likely to add LT3 therapy when surveyed in December (OR 1.6, CI 1.1–2.5, *p* = 0.023) (see Table 5). In univariate analyses, choice of therapy over time also appeared to be influenced by years in practice (*p* = 0.01), country of practice (*p* = 0.0077) and specialty (*p* = 0.025). When multivariate analyses incorporating time of survey, years in practice, country of practice, and specialty were performed, respondents were more likely to prescribe LT3 in December (*p* = 0.04), those in practice for 11–20 years were more likely to increase LT4 dosage (*p* = 0.025), those practicing in North America were more likely to prescribe LT3 (*p* = 0.003) and surgeons were more likely to increase LT4 dosage (*p* = 0.028) (see Table 6).

**Table 5:** Univariate analysis of the effect of timing on physician prescribing continued LT4 vs. increasing LT4 vs. adding LT3 to LT4 vs. replacing LT4 with T3-containing therapy.

**Time**	**Grouping of therapeutic options**	**Odds ratio**	**95% Confidence interval**		***P*-value**
March (Feb = ref)	1 vs. 2	1.03	0.8	1.4	0.83
	1 vs. 3	1.1	0.7	1.7	0.70
	1 vs. 4	0.98	0.6	1.6	0.94
Dec (Feb = ref)	1 vs. 2	1.3	0.98	1.8	0.054
	**1 vs. 3**	**1.6**	**1.1**	**2.5**	**0.023**
	1 vs. 4	1.6	0.97	2.5	0.062
December (Feb-Mar = ref)	**1 vs. 2**	**1.3**	**1.003**	**1.8**	**0.048**
	**1 vs. 3**	**1.6**	**1.1**	**2.3**	**0.022**
	**1 vs. 4**	**1.6**	**1.01**	**2.5**	**0.045**

*Continuing LT4 = therapeutic group 1 (reference), Increasing LT4 = therapeutic group 2, Adding LT3 to same or reduced LT4 = group 3, Replacing LT4 with DTE or LT3 = group 4. The options in bold font have significant p-values*.

**Table 6:** Multivariate analysis of the effect of timing on physician prescribing continued LT4 vs. increasing LT4 vs. adding LT3 to LT4 vs. replacing LT4 with T3-containing therapy.

**Timing and Physician Characteristics**		**Treatment group**	**Adjusted OR**	**95% confidence limits**	
Time (Feb = ref)	Dec	2 vs. 1	1.3	0.95	1.8
	**Dec**	**3 vs. 1**	**1.5**	**1.0**	**2.3**
	Dec	4 vs. 1	1.4	0.87	2.3
	Mar	2 vs. 1	1.1	0.76	1.5
	Mar	3 vs. 1	1.1	0.72	1.7
	Mar	4 vs. 1	0.92	0.55	1.5
Number of years in practice (in training = ref)	<5 years	2 vs. 1	1.7	0.65	4.3
	<5 years	3 vs. 1	1.8	0.54	6.2
	<5 years	4 vs. 1	2.4	0.55	10.1
	5–10 years	2 vs. 1	1.7	0.67	4.1
	5–10 years	3 vs. 1	1.5	0.46	4.8
	5–10 years	4 vs. 1	1.5	0.36	6.1
	**11–20 years**	**2 vs. 1**	**2.7**	**1.1**	**6.5**
	11–20 years	3 vs. 1	2.4	0.78	7.4
	11–20 years	4 vs. 1	2.3	0.58	8.9
	>20 years	2 vs. 1	2.0	0.84	4.6
	>20 years	3 vs. 1	1.1	0.35	3.2
	>20 years	4 vs. 1	1.5	0.39	5.6
Country of practice (other = ref)	North America	2 vs. 1	0.9	0.71	1.2
	**North America**	**3 vs. 1**	**1.8**	**1.2**	**2.6**
	North America	4 vs. 1	1.5	0.95	2.3
Specialty (internist or primary care physician = ref)	Endocrinologist	2 vs. 1	2.0	0.68	5.9
	Endocrinologist	3 vs. 1	1.1	0.27	4.3
	Endocrinologist	4 vs. 1	1.4	0.25	7.5
	**Surgeon**	**2 vs. 1**	**3.9**	**1.2**	**13.0**
	Surgeon	3 vs. 1	1.1	0.22	5.2
	Surgeon	4 vs. 1	5.3	0.81	34.3
	Nuclear medicine physician	2 vs. 1	2.9	0.83	10.2
	Nuclear medicine physician	3 vs. 1	0.77	0.14	4.1
	Nuclear medicine physician	4 vs. 1	3.1	0.44	22.1
	Other	2 vs. 1	1.6	0.44	5.8
	Other	3 vs. 1	0.56	0.10	3.1
	Other	4 vs. 1	3.2	0.45	23.1

OR and CI indicated in bold font are significant.

*Continuing LT4 = therapeutic group 1 (reference), Increasing LT4 = therapeutic group 2, Adding LT3 to same or reduced LT4 = group 3, Replacing LT4 with DTE or LT3 = group 4*.

## Discussion

Current guidelines for treatment of hypothyroidism consider LT4 to be standard of care, and the accumulated studies of combination therapy have not shown a benefit of combination therapy. Although a proportion of patients are dissatisfied with LT4, high quality studies have not yet been performed to determine whether careful, individualized LT4 dose titration may improve the symptoms of some of these patients. Current media attention to combination therapy makes it challenging to determine the relative impact of media coverage or true patient preference on patient requests for combination therapy. This analysis shows that physicians were more likely to prescribe LT3, either added to the same LT4 dose or added to a reduced LT4 dose, when surveyed in December 2017 compared with February and March 2017. Physicians practicing in North America were also more likely to prescribe such therapy compared with those practicing in other regions.

Considering the greater prescribing of LT3 in late 2017, compared with earlier in the year, other studies have reported data about prescribing patterns at various points in time. An observational study conducted in Scotland showed that 400 out of 34,355 patients (0.11%) had been prescribed LT3 during the period 1997–2014 (31). A survey about the treatment of hypothyroidism conducted in 2013 found that 0.8% of physicians would routinely use combination therapy for treating hypothyroidism, whereas 3.6% would use such therapy in a patient with persistent symptoms (32). A study conducted 3 years later in 2016 also showed that 4.2% of physicians would prescribe combination therapy for a patient with persistent symptoms consistent with hypothyroidism (33). However, although these latter two studies used the same survey instrument, it is difficult to utilize these data to determine trends as one study surveyed physicians practicing primarily in America and Europe and the respondents were primarily Endocrinologists (32), whereas the other survey queried mostly primary care physicians and was conducted in India (33). The finding that 3.6–4.2% of physicians were willing to prescribe combination therapy in both these studies contrasts markedly with the present findings that up to 47% of physicians would add LT3 therapy while reducing the LT4 dose, depending on the specific patient scenario, and that up to 38% would add LT3 therapy while maintaining the LT4 dose, again depending upon the patient characteristics. An observational study derived from pharmacy data did show that following the publication of the European Thyroid Association Guidelines (1), which stated that combination therapy could be considered under specific circumstances, there was a trend for increasing numbers of LT3 and DTE prescriptions to be received at a specific pharmacy in Denmark (34).

The results of the current survey show that approximately one third of physicians treating patients with hypothyroidism are willing in theory to prescribe therapies other than LT4. This is despite the fact that ATA guidelines for the treatment of hypothyroidism conclude that there is insufficient evidence to support prescribing T3-containing therapies (2, 4), but in keeping with more recent recommendations from British and Italian Societies (20–22). It is difficult to identify original published data that might account for the current willingness to prescribe combination therapy, and published data about preference or improvement of symptoms with such therapy is entirely from uncontrolled (23), sparsely documented (25) or small studies (24). It has become increasing common to involve patients in their own care and to incorporate joint physician-patient decision-making in the management of many conditions. It is possible that this management style, combined with increased attention to the possibilities of combination therapy in the media, social media, and patient support groups, has led to willingness to consider this therapy. Local prescribing patterns and interaction with pharmaceutical companies may also be influential.

If combination therapy is being more frequently prescribed, it is important to consider the potential risks, as well as the potential benefits. There is relatively little data available about the potential risks of combination therapy (35). This is, in part, because most studies of combination therapy are of short duration, with only one lasting a full year (5–17). One recent observational study did not identify an increased risk of atrial fibrillation or fractures with a median duration of LT3 therapy of 10.9 years (31). However, there was an increased risk of new prescriptions for antipsychotic medications and a trend for increased prescriptions of new anti-depressant medications with combination therapy.

When considering regional variations in prescribing combination therapy, the finding that combination therapy was considered more in North America than other regions was surprising, given that, in general, guidelines from North America recommend against such treatment (2, 4). It is possible that this simply reflects that most guidelines are widely disseminated throughout all geographic regions, as has been shown for ATA guidelines (36), and physicians may be particularly influenced not by guidelines from their region, but by the most recent guidelines. Unexplained variation in practice patterns has been shown for other thyroid disorders, such as thyroid cancer (37) and subclinical hypothyroidism (38, 39).

There are several limitations of our study. The number of respondents at each of our time points was relatively small, represented a small percentage of the ATA membership, and was smaller at each successive time point. The patient scenarios were presented in the same order to all physicians, rather than in a random order, making it harder to correct for an effect of order. In addition, responses about the management of theoretical patients may not reflect what a physician would do when faced with a real patient. We also did not ask physicians whether they prescribed combination therapy to patients in their own practice or when they had last prescribed combination therapy in their own practice. We also do not have data available about the number of LT3 prescriptions actually written in the US or other countries over recent years or the availability of LT3 within various insurance prescription plans or within the various countries.

In summary, this study shows that some physicians are willing to prescribe combination therapy to patients with hypothyroidism. Prescribing patterns are affected by the characteristics of the patient and the characteristics of the physician, and these prescribing patterns may be changing over time. Given that little new evidence has accrued, this trend may be due to greater consideration of patient preferences. Better studies of physiologic doses of combination therapy that rigorously examine patient preferences, patient-reported outcomes, and quality of life are clearly needed. Without such studies, authors of future hypothyroidism treatment guidelines will face a substantial quandary as to how to weight patient preferences, and the physician prescribing patterns seen in this analysis, with the negative results of combination therapy trials.

## Author Contributions

JJ designed and conducted the study. NS and ET designed and analyzed the study.

### Conflict of Interest Statement

The authors declare that the research was conducted in the absence of any commercial or financial relationships that could be construed as a potential conflict of interest.
