# Supplementary material for: Short-Term Time Trends in Prescribing Therapy for Hypothyroidism: Results of a Survey of American Thyroid Association Members
Source: Front Endocrinol (Lausanne). 2019 Jan 30;10:31. doi: 10.3389/fendo.2019.00031 (PMC6363658; doi:10.3389/fendo.2019.00031)
Supplement: Supplementary file 1 [file Table_1.DOCX]

**Survey Questions Regarding Combination Therapy**

1. **Do you prescribe and adjust levothyroxine therapy for patients with hypothyroidism?**
2. **If yes, please complete rest of survey**
3. **If no, thank you and you do not need to complete survey**

**Please answer some questions about yourself and your practice**

1. How many years have you been in practice?
   1. Currently in training
   2. <5 years
   3. 5-10 years
   4. 10-20 years
   5. > 20 years
2. Where do you practice?
   1. North America
      1. Please identify country
   2. South America
      1. Please identify country
   3. Europe
      1. Please identify country
   4. Asia
      1. Please identify country
   5. Other
      1. Please identify country
3. Which best describes your specialty?
   1. Endocrinologist
   2. Surgeon
   3. Nuclear Medicine physician
   4. Internist or primary care physician
   5. Other
      1. Please identify

**Please answer the following questions about patients being treated for hypothyroidism. All patients have normal vital signs, are being treated for overt hypothyroidism, have been taking levothyroxine for at least 5 years, and are fully adherent to their therapy. None of the women are planning a pregnancy.**

1. A 29-year old woman with Hashimoto’s hypothyroidism is taking 100 mcg of levothyroxine. She feels well. Physical examination suggests she is clinically euthyroid. Her BMI and laboratory values are below.

| **Parameter** | **Patient value** | **Normal range** |
| --- | --- | --- |
| BMI (kg/m2) | 25 | 18.5 to 24.9 |
| TSH (mIU/L) | 2.2 | 0.4-4.0 |
| Free T4 (ng/dL) | 1.3 | 0.8-1.8 |
| Total T3 (ng/dL) | 120 | 80-180 |

Which would you do?

- 1. Continue current levothyroxine
  2. Increase levothyroxine dose
  3. Add 2.5 mcg liothyronine (Cytomel) twice daily and reduce levothyroxine
  4. Add 2.5 mcg liothyronine (Cytomel) twice daily to current levothyroxine
  5. Replace levothyroxine with thyroid extract (e.g. armour thyroid)
  6. Replace levothyroxine with liothyronine (Cytomel) as single therapy

1. A 29-year old woman with Hashimoto’s hypothyroidism is taking 100 mcg of levothyroxine. She is frustrated because she is gaining weight despite regular exercise, is tired throughout the day, and has poor memory and work performance. Physical examination suggests she is clinically euthyroid. Her BMI and laboratory values are below.

| **Parameter** | **Patient value** | **Normal range** |
| --- | --- | --- |
| BMI (kg/m2) | 25 | 18.5 to 24.9 |
| TSH (mIU/L) | 2.2 | 0.4-4.0 |
| Free T4 (ng/dL) | 1.3 | 0.8-1.8 |
| Total T3 (ng/dL) | 120 | 80-180 |

Which would you do?

1. Continue current levothyroxine
2. Increase levothyroxine dose
3. Add 2.5 mcg liothyronine (Cytomel) twice daily and reduce levothyroxine
4. Add 2.5 mcg liothyronine (Cytomel) twice daily to current levothyroxine
5. Replace levothyroxine with thyroid extract (e.g. armour thyroid)
6. Replace levothyroxine with liothyronine (Cytomel) as single therapy
7. A 29-year old woman with Hashimoto’s hypothyroidism is taking 100 mcg of levothyroxine. She is frustrated because she is gaining weight despite regular exercise, is tired throughout the day, and has poor memory and work performance. Physical examination suggests she is clinically euthyroid. Her BMI and laboratory values are below.

| **Parameter** | **Patient value** | **Normal range** |
| --- | --- | --- |
| BMI (kg/m2) | 25 | 18.5 to 24.9 |
| TSH (mIU/L) | 3.9 | 0.4-4.0 |
| Free T4 (ng/dL) | 1.3 | 0.8-1.8 |
| Total T3 (ng/dL) | 120 | 80-180 |

Which would you do?

1. Continue current levothyroxine
2. Increase levothyroxine dose
3. Add 2.5 mcg liothyronine (Cytomel) twice daily and reduce levothyroxine
4. Add 2.5 mcg liothyronine (Cytomel) twice daily to current levothyroxine
5. Replace levothyroxine with thyroid extract (e.g. armour thyroid)
6. Replace levothyroxine with liothyronine (Cytomel) as single therapy
7. A 29-year old woman with Hashimoto’s hypothyroidism is taking 100 mcg of levothyroxine. She is frustrated because she is gaining weight despite regular exercise, is tired throughout the day, and has poor memory and work performance. Physical examination suggests she is clinically euthyroid. Her BMI and laboratory values are below.

| **Parameter** | **Patient value** | **Normal range** |
| --- | --- | --- |
| BMI (kg/m2) | 25 | 18.5 to 24.9 |
| TSH (mIU/L) | 2.2 | 0.4-4.0 |
| Free T4 (ng/dL) | 1.3 | 0.8-1.8 |
| Total T3 (ng/dL) | 75 | 80-180 |

Which would you do?

1. Continue current levothyroxine
2. Increase levothyroxine dose
3. Add 2.5 mcg liothyronine (Cytomel) twice daily and reduce levothyroxine
4. Add 2.5 mcg liothyronine (Cytomel) twice daily to current levothyroxine
5. Replace levothyroxine with thyroid extract (e.g. armour thyroid)
6. Replace levothyroxine with liothyronine (Cytomel) as single therapy
7. A 29-year old woman with Hashimoto’s hypothyroidism is taking 100 mcg of levothyroxine. She is frustrated because she is gaining weight despite regular exercise, is tired throughout the day, and has poor memory and work performance. Physical examination suggests she is clinically euthyroid. Her BMI and laboratory values are below.

| **Parameter** | **Patient value** | **Normal range** |
| --- | --- | --- |
| BMI (kg/m2) | 25 | 18.5 to 24.9 |
| TSH (mIU/L) | 3.9 | 0.4-4.0 |
| Free T4 (ng/dL) | 1.3 | 0.8-1.8 |
| Total T3 (ng/dL) | 75 | 80-180 |

Which would you do?

1. Continue current levothyroxine
2. Increase levothyroxine dose
3. Add 2.5 mcg liothyronine (Cytomel) twice daily and reduce levothyroxine
4. Add 2.5 mcg liothyronine (Cytomel) twice daily to current levothyroxine
5. Replace levothyroxine with thyroid extract (e.g. armour thyroid)
6. Replace levothyroxine with liothyronine (Cytomel) as single therapy
7. A 29-year old woman with Hashimoto’s hypothyroidism is taking 100 mcg of levothyroxine. She is frustrated because she is gaining weight despite regular exercise, is tired throughout the day, and has poor memory and work performance. She requests “combination” therapy. Physical examination suggests she is clinically euthyroid. Her BMI and laboratory values are below.

| **Parameter** | **Patient value** | **Normal range** |
| --- | --- | --- |
| BMI (kg/m2) | 25 | 18.5 to 24.9 |
| TSH (mIU/L) | 2.2 | 0.4-4.0 |
| Free T4 (ng/dL) | 1.3 | 0.8-1.8 |
| Total T3 (ng/dL) | 75 | 80-180 |

Which would you do?

1. Continue current levothyroxine
2. Increase levothyroxine dose
3. Add 2.5 mcg liothyronine (Cytomel) twice daily and reduce levothyroxine
4. Add 2.5 mcg liothyronine (Cytomel) twice daily to current levothyroxine
5. Replace levothyroxine with thyroid extract (e.g. armour thyroid)
6. Replace levothyroxine with liothyronine (Cytomel) as single therapy
7. A 29-year old woman with post-surgical hypothyroidism following a total thyroidectomy is taking 100 mcg of levothyroxine regularly. She is frustrated because she is gaining weight despite regular exercise, is tired throughout the day, and has poor memory and work performance. She requests “combination” therapy. Physical examination suggests she is clinically euthyroid. Her BMI and laboratory values are below.

| **Parameter** | **Patient value** | **Normal range** |
| --- | --- | --- |
| BMI (kg/m2) | 25 | 18.5 to 24.9 |
| TSH (mIU/L) | 2.2 | 0.4-4.0 |
| Free T4 (ng/dL) | 1.3 | 0.8-1.8 |
| Total T3 (ng/dL) | 75 | 80-180 |

Which would you do?

1. Continue current levothyroxine
2. Increase levothyroxine dose
3. Add 2.5 mcg liothyronine (Cytomel) twice daily and reduce levothyroxine
4. Add 2.5 mcg liothyronine (Cytomel) twice daily to current levothyroxine
5. Replace levothyroxine with thyroid extract (e.g. armour thyroid)
6. Replace levothyroxine with liothyronine (Cytomel) as single therapy
7. A 29-year old woman with Hashimoto’s hypothyroidism is taking 100 mcg of levothyroxine regularly. She is frustrated because she is gaining weight despite regular exercise, is tired throughout the day, and has poor memory and work performance. She tells you that a previous physician added T3 to her regimen and she felt better. She requests a return to “combination” therapy. Physical examination suggests she is clinically euthyroid. Her BMI and laboratory values are below.

| **Parameter** | **Patient value** | **Normal range** |
| --- | --- | --- |
| BMI (kg/m2) | 25 | 18.5 to 24.9 |
| TSH (mIU/L) | 2.2 | 0.4-4.0 |
| Free T4 (ng/dL) | 1.3 | 0.8-1.8 |
| Total T3 (ng/dL) | 75 | 80-180 |

Which would you do?

1. Continue current levothyroxine
2. Increase levothyroxine dose
3. Add 2.5 mcg liothyronine (Cytomel) twice daily and reduce levothyroxine
4. Add 2.5 mcg liothyronine (Cytomel) twice daily to current levothyroxine
5. Replace levothyroxine with thyroid extract (e.g. armour thyroid)
6. Replace levothyroxine with liothyronine (Cytomel) as single therapy
7. A 29-year old man with Hashimoto’s hypothyroidism is taking 100 mcg of levothyroxine. He is frustrated because he is gaining weight despite regular exercise, is tired throughout the day, and has poor memory and work performance. He requests “combination” therapy. Physical examination suggests he is clinically euthyroid. His BMI and laboratory values are below.

| **Parameter** | **Patient value** | **Normal range** |
| --- | --- | --- |
| BMI (kg/m2) | 25 | 18.5 to 24.9 |
| TSH (mIU/L) | 2.2 | 0.4-4.0 |
| Free T4 (ng/dL) | 1.3 | 0.8-1.8 |
| Total T3 (ng/dL) | 75 | 80-180 |

Which would you do?

1. Continue current levothyroxine
2. Increase levothyroxine dose
3. Add 2.5 mcg liothyronine (Cytomel) twice daily and reduce levothyroxine
4. Add 2.5 mcg liothyronine (Cytomel) twice daily to current levothyroxine
5. Replace levothyroxine with thyroid extract (e.g. armour thyroid)
6. Replace levothyroxine with liothyronine (Cytomel) as single therapy
7. A 29-year old woman with Hashimoto’s hypothyroidism is taking 100 mcg of levothyroxine. She is frustrated because she is gaining weight despite regular exercise, is tired throughout the day, and has poor memory and work performance. She previously participated in a study that showed that she had a genetic problem with converting T4 to T3. She requests “combination” therapy. Physical examination suggests she is clinically euthyroid. Her BMI and laboratory values are below.

| **Parameter** | **Patient value** | **Normal range** |
| --- | --- | --- |
| BMI (kg/m2) | 25 | 18.5 to 24.9 |
| TSH (mIU/L) | 2.2 | 0.4-4.0 |
| Free T4 (ng/dL) | 1.3 | 0.8-1.8 |
| Total T3 (ng/dL) | 75 | 80-180 |

Which would you do?

1. Continue current levothyroxine
2. Increase levothyroxine dose
3. Add 2.5 mcg liothyronine (Cytomel) twice daily and reduce levothyroxine
4. Add 2.5 mcg liothyronine (Cytomel) twice daily to current levothyroxine
5. Replace levothyroxine with thyroid extract (e.g. armour thyroid)
6. Replace levothyroxine with liothyronine (Cytomel) as single therapy
7. A 59-year old woman with Hashimoto’s hypothyroidism is taking 100 mcg of levothyroxine. She is frustrated because she is gaining weight despite regular exercise, is tired throughout the day, and has poor memory and work performance. She requests “combination” therapy. Physical examination suggests she is clinically euthyroid. Her BMI and laboratory values are below.

| **Parameter** | **Patient value** | **Normal range** |
| --- | --- | --- |
| BMI (kg/m2) | 25 | 18.5 to 24.9 |
| TSH (mIU/L) | 2.2 | 0.4-4.0 |
| Free T4 (ng/dL) | 1.3 | 0.8-1.8 |
| Total T3 (ng/dL) | 75 | 80-180 |

Which would you do?

1. Continue current levothyroxine
2. Increase levothyroxine dose
3. Add 2.5 mcg liothyronine (Cytomel) twice daily and reduce levothyroxine
4. Add 2.5 mcg liothyronine (Cytomel) twice daily to current levothyroxine
5. Replace levothyroxine with thyroid extract (e.g. armour thyroid)
6. Replace levothyroxine with liothyronine (Cytomel) as single therapy
7. A 29-year old woman with Hashimoto’s hypothyroidism is taking 100 mcg of levothyroxine. She is frustrated because she is gaining excessive weight despite regular exercise, is tired throughout the day, and has poor memory and work performance. She requests “combination” therapy. Physical examination suggests she is clinically euthyroid. Her BMI and laboratory values are below.

| **Parameter** | **Patient value** | **Normal range** |
| --- | --- | --- |
| BMI (kg/m2) | 32 | 18.5 to 24.9 |
| TSH (mIU/L) | 2.2 | 0.4-4.0 |
| Free T4 (ng/dL) | 1.3 | 0.8-1.8 |
| Total T3 (ng/dL) | 75 | 80-180 |

Which would you do?

1. Continue current levothyroxine
2. Increase levothyroxine dose
3. Add 2.5 mcg liothyronine (Cytomel) twice daily and reduce levothyroxine
4. Add 2.5 mcg liothyronine (Cytomel) twice daily to current levothyroxine
5. Replace levothyroxine with thyroid extract (e.g. armour thyroid)
6. Replace levothyroxine with liothyronine (Cytomel) as single therapy
7. A 59-year old woman with Hashimoto’s hypothyroidism and steroid-induced osteoporosis is taking 100 mcg of levothyroxine. She is frustrated because she is gaining weight despite regular exercise, is tired throughout the day, and has poor memory and work performance. She requests “combination” therapy. Physical examination suggests she is clinically euthyroid. Her BMI and laboratory values are below.

| **Parameter** | **Patient value** | **Normal range** |
| --- | --- | --- |
| BMI (kg/m2) | 25 | 18.5 to 24.9 |
| TSH (mIU/L) | 2.2 | 0.4-4.0 |
| Free T4 (ng/dL) | 1.3 | 0.8-1.8 |
| Total T3 (ng/dL) | 75 | 80-180 |

Which would you do?

1. Continue current levothyroxine
2. Increase levothyroxine dose
3. Add 2.5 mcg liothyronine (Cytomel) twice daily and reduce levothyroxine
4. Add 2.5 mcg liothyronine (Cytomel) twice daily to current levothyroxine
5. Replace levothyroxine with thyroid extract (e.g. armour thyroid)
6. Replace levothyroxine with liothyronine (Cytomel) as single therapy
